# Parkinson's disease: From genetics to molecular dysfunction and targeted therapeutic approaches

**DOI:** 10.1016/j.gendis.2021.12.015

**Published:** 2022-02-05

**Authors:** Yue Huang, Jun Wei, Antony Cooper, Margaret J. Morris

**Affiliations:** a China National Clinical Research Center for Neurological Diseases, Beijing Tiantan Hospital, Capital Medical University, Beijing 100070, China; b Department of Neurology, Beijing Tiantan Hospital, Capital Medical University, Beijing 100070, China; c Department of Pharmacology, School of Medical Sciences, Faculty of Medicine & Health, UNSW, Sydney, NSW 2052, Australia; d The Garvan Institute of Medical Research, Sydney, NSW 2010, Australia; e St Vincent's Clinical School, Faculty of Medicine & Health, and School of Biotechnology and Biomolecular Sciences, Faculty of Science, UNSW, Sydney, NSW 2052, Australia

**Keywords:** Drug discovery, Genetics, Molecular function, Parkinson's disease, Quantitative traits

## Abstract

Parkinson's disease (PD) is the most common neurodegenerative movement disorder in the elderly. As the pathogenesis of PD is still not fully understood, medications with the capacity of halting the disease progression are currently unavailable. The discovery of genes that are causative for, or increase susceptibility to PD is pivotal for the development of novel therapeutic approaches, as they are critical for the onset of PD and the molecular pathways underlying its pathogenesis. By reviewing relevant data, we discuss causative genes, and those associated with PD susceptibility and quantitative traits. Through Gene Ontology database and STRING analysis, we emphasize the roles of inorganic cation transmembrane transport pathways and hypothalamic pituitary thyroid axis, in addition to the established roles of inflammation/oxidative stress and mitochondrial dysfunction in the pathogenesis of PD. It is hoped these insights 1) untangle the clinical complex presentations of PD, 2) reveal the interwoven molecular network leading to PD, and 3) identify critical molecular targets to facilitate novel PD drug discovery, with a view to providing improved consultation and personalized medicine for patients with PD in the future.

## Introduction

Parkinson's disease (PD) is the second most common age-related neurodegenerative disease worldwide, affecting over ten million people in the world. The prevalence of PD increases with age, affecting 0.1%–0.2% of the population across all ages, 1% of those over 60 years, and the disease costs over 51.9 billion dollars annually.^1^^,^^2^ An increased understanding of this disease is critically important, particularly in those countries with an ageing population. Many recent studies have identified genetic variants associated with PD. This review will focus on the common PD risk genetic loci and single nucleotide polymorphisms (SNPs) identified in European Caucasian and Asian populations and their impacts on the precision medicine of PD.

Although the pathogenesis of PD is not yet fully understood, striatal dopamine deficiency due to the degeneration of dopaminergic neurons of the substantia nigra pars compacta has been recognized as a PD hallmark.^3^^,^^4^ The substantia nigra appears depigmented macroscopically due to the death of neuromelanin containing dopaminergic neurons, and there are two distinct microscopic features for the pathological diagnosis of PD: intracellular α-synuclein aggregations and dopaminergic cell degeneration.^5^^,^^6^ α-Synuclein can exist as a small soluble monomer which can form oligomers or larger protein aggregates, that are components of Lewy bodies and Lewy neurites in dopaminergic neurons.^6^^,^^7^ Due to the dopaminergic neuronal dysfunction and cell death, there is insufficient dopamine in the striatum, which affects the initiation of movement,^4^^,^^5^ that in turn accounts for the movement symptoms displayed by patients. Therefore, replacing striatal dopamine through medications such as l-dopa is an effective symptomatic treatment,^3^^,^^5^ but all existing symptomatic therapies for PD (including l-dopa) do not target the underlying molecular mechanisms of the disease, and have little to no impact on disease progression.

The clinical presentations of PD are asymmetrical and develop progressively.^5^ Although the common motor symptoms are bradykinesia, resting tremor, muscle rigidity and postural instability, there are a variety of non-motor presentations including neuropsychiatric symptoms (e.g., depression, anxiety, sleep disorders), autonomic dysfunction (e.g., gastrointestinal symptoms of constipation), as well as sensory symptoms (e.g., olfactory dysfunction), which may present earlier than motor symptoms.^8^ Other symptoms such as pain, fatigue, weight changes, and dementia can also occur, usually in late stages of PD.^7^^,^^9^ Age is the major risk factor for PD, but risk is also attributed to environmental factors that include dairy products, pesticides, methamphetamine, and brain trauma.^10^ The genetic component of PD has received more interest following GWAS studies that implicate familial PD genes as risk loci for sporadic PD.^11^ This builds the foundation of identifying the SNPs and associated genes to advance our understanding of the molecular mechanisms that confer increased risk of PD.

## PD causative genes

The first PD causative mutation was discovered in the *SNCA* gene in Italian and Greek kindreds in 1997.^12^ Subsequently, many other PD causative genes were identified from linkage analysis by segregating genes from monogenetic PD-affected families (Table 1). PD causative genes follow either autosomal dominant or recessive inherited patterns, mainly reflecting gain or loss of its correspondent molecular functions respectively.

**Table 1:** PD causative genes.

PARK	Gene	Loci	Protein cellular distribution and function	Mutations	Inheritance	Clinical Phenotypes
*PARK1*	*SNCA*	4q21-23	α-synuclein^17^: presynaptic signaling and membrane trafficking.^93^	A53T, A30P,^17^^,^^94^ A18T, A29S, G46L, H50G, G51A^95^	AD	Young-onset and late-onset hereditary Lewy body PD^96^
*PARK2*	*PRKN*	6q25.2-q27	Parkin RBR E3 ubiquitin protein ligase: regulate the autophagic degradation of mitochondria.^54^	Deletions of exons 1, 4 and 5, and P113Xfs, R275W, G430D and R33X^97^	AR	Young-onset PD (mean onset of PD at 32 y.o.)^98^
*PARK3(putative)*	*Unknown*	2p13	–	Not identified	AD	Late onset idiopathic Lewy body PD.^99^
*PARK4*	*SNCA*	4p22.1	α-synuclein^17^: presynaptic signaling and membrane trafficking.^93^	duplication and triplication.^18^^,^^19^	AD	Young-onset and late-onset hereditary Lewy body PD^96^
*PARK5(putative)*	*UCHL1*	4p13	Ubiquitin C-Terminal Hydrolase L1: processing ubiquitinated proteins and ubiquitin precursors.^100^	Unconfirmed	AD	Late onset PD(mean onset of PD at 50 y.o.)^100^
*PARK6*	*PINK1*	1p36.12	PTEN induced kinase 1: mitochondria degradation^44^	Deletions in exon 1, 5, and 7^51,97^, g.16378G > A, c.1488 + 1G > A^51^ and W90Xfs^97^	AR	Young-onset (mean age at 31.6 y.o.), slowly progressive levodopa-responsive PD.^51^
*PARK7*	*DJ-1*	1p36.23	Parkinsonism associated deglycase: cell protection from toxic stresses.^101^	M26I, G78G, R98Q, R98R, D149A, A167A and InsA+120.^102^	AR	Young-onset PD.^102^
*PARK8*	*LRRK2*	12q12	Dardarin: GTPase and kinase.^103^	G2019S,^104^ R1441G, R1441 C/H, Y1699C, R1628P, G2385R and I2020T.^105^	AD	Mean onset of PD at 58·1 y.o, hereditary (mutations present in 10% patients) and idiopathic (mutations present in 4% of patients) Lewy body PD.^7^^,^^33^
*PARK9 (parkinsonism causative)*	*ATP13A2*	1p36.13	Lysosomal type 5 ATPase: maintain intracellular cation homeostasis and neuronal integrity^106^	Deletion in exon 26, 22-bp duplication (1632_1653dup22), 2-bp insertion (1103insGA),^107^ 1306+5G-A,^108^ G504R,^109^ M810R,^110^ G877R.^111^	AR	Kufor-Rakeb syndrome: early onset idiopathic Parkinsonism associated with mask-like face, rigidity and bradykinesia, spasticity, supranuclear gaze palsy and dementia.^112^
*PARK10(putative)*	*Unknown*	1p32	–	Unconfirmed	AD	Late onset PD (mean onset of PD at 65.8 y.o.)^113^
*PARK11(putative)*	*GIGYF2(confronted)*	2q37.1	GRB10-interacting GYF protein 2: repressing translation initiation^114^	Deletion L1230_Q1237del, N478T, H1992R^115^	AD	Late onset idiopathic PD^116^
*PARK12(putative)*	*Unknown*	Xq21-q25	–	Unconfirmed	X-linked	Late onset PD^117^
*PARK13(putative)*	*HTRA2(confronted)*	2p13.1	HtrA Serine Peptidase 2: proteolytic activity and promotes apoptosis^118^	G399S and A141S^119^	AD	Late onset PD (mean onset of PD at 57.3 y.o.) in German population
*PARK14 (parkinsonism causative)*	*PLA2G6*	22q13.1	Phospholipase A2 Group VI: phospholipid remodeling for cellular membrane homeostasis^120^	R741Q and R747W^121^	AR	Parkinsonism, dystonia and cognitive decline^121^
*PARK15 (parkinsonism causative)*	*FBXO7*	22q12.3	F-box only protein 7: mediating the ubiquitination and proteasomal degradation^122^	A498Stop, T22M, and splice-site IVS7 + 1G/T^123^	AR	Early onset levodopa-responsive Parkinsonian-pyramidal syndrome^124^
*PARK16(putative)*	*Unknown*	1q32	–	unconfirmed	unconfirmed	unconfirmed
*PARK17*	*VPS35*	16q11.2	VPS35 retromer complex component: vesicle transport and membrane-protein recycling.^125^^,^^126^	A620A^127^ and P316S^126^	AD	Late onset, hereditary PD (mean onset of PD at 53 y.o.).^127^
*PARK18*	*EIF4G1*	3q26-q28	Eukaryotic translation initiation factor 4 gamma 1: involved in mRNA translation.^128^	A1205H, A502V, G686C, S1164A and A1197T.^129^	AD	Late onset PD (mean onset of PD at 52–64 y.o. for each mutation).
*PARK19*	*DNAJC6*	1p31.3	DnaJ Heat Shock Protein Family (Hsp40) Member C6: clathrin-mediated endocytosis (by similarity)	Q734X^130^ Q789X^131^ and R927G^132^	AR	Juvenile onset or early adult-onset PD^132^^,^^133^
*PARK20*	*SYNJ1*	21q22.11	Synaptojanin-1: clathrin-mediated endocytosis (by similarity)	R258Q,^134^^,^^135^ R459P^136^	AR	Early onset PD in an Iranian and an Italian population^134^
*PARK21*	*DNAJC13*	3q22.1	DnaJ heat shock protein family (Hsp40) member C13: endosomal membrane regulation.^137^	A855S^138^	AD	Late onset PD (mean onset of PD at 67 y.o.)^138^
*PARK22*	*CHCHD2*	7p11.2	Coiled-coil-helix-coiled-coil-helix domain containing 2: mitochondrial respiration^139^	A32T, P34L, and I80V^139^	AD	Early onset PD^139^
*PARK23*	*VPS13C*	15q22.2	Vacuolar protein sorting-associated protein 13C: Maintaining mitochondrial transmembrane potential.^131^	Truncating mutations^131^	AR	Rapidly progressive PD in Turkish and French population^131^

### PD causative genes with autosomal dominant inherence

Although multiple genes have been shown causative for PD (Table 1), two important PD causative genes identified so far are *SNCA* and *LRRK2*. *SNCA* encodes α-synuclein, a major component of pathological hallmark of Lewy bodies in PD,^13^ while mutations in *LRRK2* are the most common indicators of inherited PD.^14^^,^^15^ Over time, more PD causative genes with autosomal dominant inherence were identified (Table 1), but none has overtaken the importance of *SNCA* or *LRRK2* from pathological or genetic perspective, as outlined below.

α-Synuclein is a 140 amino acid presynaptic protein with multiple conformations and exists in many oligomeric states in a dynamic equilibrium.^16^ Mutant α-synuclein changes its conformation making it prone to form aggregates and Lewy bodies. Amongst the *SNCA* mutations, the missense mutation A53T was hypothesized as disrupting the α helix and extending the β sheet structure.^12^ In addition to the Greek pedigree, A53T and other missense mutations in *SNCA* such as A30P and E46K have been identified in over 12 Mediterranean PD families.^11^ These *SNCA* missense mutations lead to structural changes in α-synuclein,^17^ in which A30P and A53T mutations form annular and pore-like protofibrils, and annular and tubular prefibrillar oligomers correspondingly, under electron microscopy, analytical ultracentrifugation and scanning transmission electron microscopy.^17^ Apart from point mutations, multiplications of the *SNCA* region lead to correspondingly elevated expression of α-synuclein, and hence cause typical and atypical PD.^11^^,^^18^ The *SNCA* genomic multiplications occur due to unequal cross-over during either intra-allelic or inter-allelic recombination or both.^19^ The multiplications appear to associate with early onset of PD,^11^ e.g., *SNCA* triplication has been identified as causing dominant early-onset PD.^20^ The dosage of *SNCA* multiplications impacts on the severity of PD and dementia presentations^19^ due to *SNCA* over-expression, that can increase α-synuclein aggregation and fibril formation.

The addition of recombinant α-synuclein fibrils to primary neurons led to the selective decreases in synaptic proteins, progressive impairments in neuronal connectivity and eventually neuron death.^21^ In addition, inoculation of recombinant α-synuclein fibrils in the striatum of mice led to pathological cell to cell α-synuclein transmission eventually resulting in dopaminergic neuronal loss in the substantia nigra accompanied by motor deficit.^22^ Thus, pathologic levels of α-synuclein can induce neuronal toxicity.

The N-terminal 32 amino acids of human α-synuclein contain cryptic mitochondrial targeting signal, which is important for α-synuclein binding to mitochondrial membrane and allows α-synuclein to be imported into mitochondria.^23^^,^^24^ Mitochondrial accumulation of α-synuclein potentially affects respiratory complex I activity, increases oxidative stress, and leads to neuronal toxicity.^23^^,^^24^ The C-terminus of α-synuclein interacts with the microtubule binding domain of tau, particularly when tau is hyperphosphorylated,^25^ and facilitates the formation of neuropathological intraneuronal filamentous inclusinons.^26^^,^^27^ Molecular functions of α-synuclein provide further pathophysiological evidence about the critical role of α-synuclein in the pathogenesis of PD.

*LRRK2* encodes a large 51-exon multi-domain protein of over 2500 amino acids.^28^ Its multiple roles include participation in vesicle sorting by mediating the endosomal-autophagic pathway and late endosomal membrane trafficking.^29^, ^30^, ^31^ Over 40 missense mutations in *LRRK2* have been identified,^11^ all of them displaying an autosomal dominant PD pattern, of which G2385R variant (rs34778348) confers a risk for people to develop PD in Asia.^32^ Studies have found the frequency and penetrance of *LRRK2* mutations vary significantly among different ethnicities.^28^ *LRRK2* mutations are found in 2% of patients with PD.^14^ Gly2019Ser is the most common *LRRK2* mutation, and is present at high frequencies mainly in amongst North African and Arabs idiopathic and hereditary PD patients at 39% and 36% correspondingly, as well as in Caucasian PD patients,^33^ but Gly2019Ser is a rare mutation in Asian populations.^11^ Other studies have also shown that the frequency and penetrance of *LRRK2* mutations vary significantly amongst different ethnicities.^28^

*LRRK2*-coded protein contains a GTPase core and kinase domain. The GTPase catalytic core regulates its kinase domain.^34^ LRRK2 phosphorylates endophilin A at S75 which regulates synaptic vesicle endocytosis and EndoA-dependent membrane tubulation.^35^ LRRK2 also phosphorylates eukaryotic initiation factor 4E-binding protein (4E-BP), which modulates the eIF4E/4E-BP pathway and stimulates eIF4E-mediated protein translation, resulting in attenuation of resistance to oxidative stress and survival of dopaminergic neuron.^36^ PD associated mutations in *LRRK2* increase its kinase activity on endophilin-A leading to initiation of endocytosis,^37^ and they can also affect protein synthesis, mitochondrial quality control and further influence neuronal viability.^38^^,^^39^ LRRK2 facilitates α-synuclein inclusion formation.^40^, ^41^, ^42^ Either mutant α-synuclein or mutant LRRK2 can block or disrupt mitophagy or delay autophagosome trafficking.^43^ Convergent mechanisms of LRRK2 and α-synuclein can act on different targets within the autophagy-lysosomal system, thus leading to PD pathogenesis.

### PD causative genes with AR inherence

PD causative genes with AR inherence often occur in PD patients with early onset. Among them, the most common mutations are in *PRKN* (previously known as *PARK2*), followed by *PINK1*,^44^ and *DJ-1*,^45^ accounting for about 18%, 15% and 0.2% of early onset PD respectively.^46^, ^47^, ^48^, ^49^ Deletions and mutations in *PRKN* gene are associated with degeneration of pigmented neurons in the substantia nigra, similar to that seen in PD, but without Lewy bodies on brain autopsy.^50^^,^^51^ However, the compound heterozygous *PRKN* or *PINK1* mutations had dopaminergic neuron loss in substantia nigra and the presence of Lewy bodies.^50^^,^^51^ The pathological differences lie in the complete or partial depletion of its molecular functions caused by mutations. *PRKN* encodes the E3 ubiquitin ligase parkin, and glycosylated α-synuclein is one of the substrates normally ubiquitinated by parkin.^52^ Therefore, the parkin mutations inhibits the degradation of α-synuclein. Moreover, parkin deficient mice do not show exacerbated α-synuclein aggregation when crossed with A53T mice,^53^ suggesting strong dominant effects of *SNCA*. Parkin contributes to mitochondrial degradation along with PINK1 and DJ-1.^54^ Mutations in these PD-AR genes have been associated with dysfunction in *PRKN* and *PARK1* mediated mitochondrial quality control through processes including mitophagy, transport, biogenesis, fission and fusion.^55^, ^56^, ^57^

## Genes associated with PD susceptibility

Since 2009, genome wide association studies (GWAS) have opened a new era to identify PD susceptibility genes via comparison between PD and controls. The first European PD GWAS analysis identified two genetic risk loci with 1713 PD cases and 3978 controls and replicated with 3361 cases and 4573 controls.^58^ The risk loci identified were *SNCA* and *MAPT*, containing risk SNPs rs2736990 and rs393152, respectively.^58^ This study also replicated *PARK16* and the SNPs rs823128 as one of the SNPs previously identified in a Japanese cohort.^58^ However, some genetic regions e.g., *MAPT* have genetic heterogeneity in different ethnicities, and the PD association with *MAPT* gene was not replicated in a Japanese population, according to the GWAS study in 2009.^59^

The meta-analysis performed by the International Parkinson Disease Genomics Consortium in 2011, involved 5 American and European cohorts and over 7 million SNPs, identified up to 11 risk loci and the most significant SNP in each locus.^60^ Referring to the genes closest to a SNP, these SNPs were: chr1:154,105,678 (*SYT11*), rs6710823 (*ACMSD*), rs2102808 (*STK39*), rs11711441 (*MCCC1/LAMP3*), chr4:911,311 (*GAK*), rs11724635 (*BST1*), rs356219 (*SNCA*), chr6:32,588,205 (*HLA-DRB5*), rs1491942 (*LRRK2*), rs12817488 (*CCDC62/HIP1R*) and rs2942168 (*MAPT*).^60^

Nalls et al's meta-analysis carried in 2014 initiated an expansion in the discovery of PD associated SNPs by involving all the up-to-date PD GWAS data in European population.^61^ The study involved over 7 million variants from 1000 Genomes Project in over 13,000 cases and over 95,000 controls. There were 26 independent risk genetic loci identified by primary meta-analysis with the GWAS summary statistics.^61^ In the replication test in a separate sample set using NeuroX genotyping array that includes over 264,000 variants, 22 out of the 26 genetic loci were replicated and 6 novel loci were identified: *SIPA1L2, INPP5F, MIR4697, GCH1, VPS13C* and *DDRGK1*.^61^ A total of 28 independent risk variants (SNPs) for PD across 24 loci were identified in that study.^61^ There is evidence suggesting interactions between risk loci eg.rs199347 associates with increased expression of *NUPL2* and decreased methylation of *GPNMB*, and rs823118 increases *RAB7L1* expression and decreases *BUCKS1* expression.^61^

In 2017, Chang et al's GWAS identified 12 risk loci, one of them being the novel locus: rs9468199.^62^ They then performed meta-analysis with their GWAS data and recent GWAS data, and identified 35 novel risk loci of which 17 loci could be replicated.^62^ On the other hand, the GWAS analysis included six East Asian regions, including mainland China, Hong Kong, Taiwan, Singapore, Malaysia, and Korea, also confirming *SNCA* and *LRRK2* as the most significant risk loci, as well as *MCCC1*, and 14 other loci reported in European studies.^63^ This finding suggested mutations in *SNCA* and *LRRK2* significantly change corresponding protein functions causing PD, while their non-coding genetic variants lead to subtle changes in protein functions, conferring risk to develop PD. While *MAPT* is reported to be a PD risk gene in Asian populations, it appears there are different genetic risk variants of *MAPT* in Asian populations compared to Caucasians.^63^^,^^64^

The 2019 meta-analysis Nalls et al performed included 17 recent GWAS datasets in European populations, involving over 37,000 cases, over 18,000 PD family cases and 1.4 million controls.^65^ Ninety PD risk SNPs involved in 78 risk loci were identified.^65^ On the other hand, the meta-analysis of recent GWAS data conducted in 2020 from mainland China, Hong Kong, Taiwan, Singapore, Malaysia and South Korea populations, identified 11 risk loci, of which 9 were previously identified in a European population: *PARK16, ITPKB, MCCC1, SNCA, FAM47E-SCARB2, DLG2, LRRK2, RIT2* and *FYN*.^66^ There were novel SNPs rs246814 and rs9638616 associated with *SV2C* and *WBSCR17* (*GALNT17*) genes respectively, in which the *SV2C* intronic SNP was subsequently replicated in the European cohort, but the *WBSCR17* associated variant did not increase PD risk in European populations.^66^ Thus, these recent studies in Asia show population genetic heterogeneity in certain PD risk genes. However, GWAS studies of PD with Asian populations are still at a relatively early stage, with a limited number of studies. Future studies are needed to explore the genetic risk factors of PD in different ethnic groups and obtain a better understanding of any common or population-specific genetic variants amongst different ancestries. These aims match those of the recently established Global Parkinson's Genetic program (GP^2^) which seeks to genotype >150,000 volunteers from Africa, Asia, Europe, and the American continent (https://parkinsonsroadmap.org/gp2/).

So far, there have been over 90 independent PD risk SNPs identified in European populations, and these could explain 16–36% of the heritable risk of PD depending on prevalence.^65^ The GWAS PD susceptibility studies have been summarized (Table 2), and replication in different populations is an essential step for susceptibility gene confirmation. However, the high heterogeneity of different genomic constructs in human ethnic groups and low effect of the SNPs could potentially result in them failing to be replicated.^61^ This resolution of genetic factors could be improved by increasing the sample size.^61^ PD risk SNPs are typically associated with a small individual risk, but they occur more frequently in the population compared to PD causative mutations, and have substantial cumulative risk.^67^ These SNPs are only associated with a small PD risk and are not useful independently in making prognosis of an individual under risk to develop PD. A polygenic risk score (PRS) was therefore introduced, which is calculated by accumulating each risk SNPs as parameters.^68^^,^^69^ This allows each individual to receive a PRS and understand their PD genetic susceptibility. Based on the currently identified risk SNPs, the PRS model could predict PD with a sensitivity of 0.628 and a specificity of 0.686.^65^ Therein, PRS combining information on additional numbers of PD risk SNPs to assess the risk for developing PD is likely the future direction of genetics of PD.

**Table 2:** PD susceptibility loci via GWAS or Meta-GWAS studies.

Timeline	Number of risk loci	Number of SNP (rs)	Number of cases *vs.* control		Population	References
			Discovery stage	Replication stage		
2009	3	3	1713 *vs*. 3978	3361 *vs*. 4573	Caucasian	GWAS^58^
2009	4	23	1078 *vs*. 2628	612 *vs.* 14,139 321 vs 1614	Japanese	GWAS^59^
2011	11	11	5333 *vs*. 12,019	7053 *vs*. 9007	Caucasian	Meta-GWAS^60^
2014	22	28	13,708 *vs*. 95,282	5353 *vs*. 5551	Caucasian	Meta-GWAS^61^
2017	35	44	GWAS: 6476 *vs*. 302,042 Meta-analysis: 13,000+ *vs*. 95,000+	5851 *vs*. 5866	Caucasian	GWAS and meta-GWAS^62^
2017	73	90	779 *vs*. 13,227	5125 *vs*. 17,604	Asian	GWAS^63^
2019	78	90	30,271 *vs*. 1,014,601	26,035 vs 403,190	Caucasian	Meta-GWAS^65^
2020	11	11	6724 *vs*. 24,851	58,533 vs 1,871,337	Asian	GWAS and meta-GWAS^66^

## Genes associated with PD quantitative traits

Compared to PD genetic susceptibility studies aimed at identifying people at risk of developing PD, genetic studies on PD quantitative traits represent another important stream to identify genetic contributions to the disease process and to further distinguish PD risk from variants affecting PD progression, as slowing/stopping progression is a major goal. PD quantitative traits include continuous variables that include onset age, motor and non-motor severity measures. Patients carrying mutant genes with AR inheritance often have a benign disease course, whereas patients carrying *SNCA* triplication often have more severe disease course compared to patients with *SNCA* duplication.^19^ Heterozygous mutations in *GBA* accounts for 2.3%–17.9% patients with PD, although *GBA* is usually not considered as a PD gene due to the incomplete penetrance of *GBA* mutations, and thus *GBA* mutations are instead frequently viewed as a strong risk factor for PD.^70^ *GBA* gene mutations have also been associated with PD symptoms severity, rate of disease progression, and age of onset.^70^

Some of the PD risk loci have been shown to be associated with the age at onset^71^ and the progression of PD in Caucasian and in Asian populations.^64^^,^^72^ Furthermore, PRS also indicates contribution to the prognosis for PD progression, which is proven to associate with the motor and cognitive functional decline among PD patients.^69^ There is evidence suggesting a higher PRS is associated with early PD onset, however, PRS was not shown to associate with amount of α-synuclein in CSF, which might suggest more SNPs are need to be identified and included in the PRS calculation.^73^ In addition, a GWAS association study with PD progression was first attempted this year to evaluate genomic contribution to the motor and non-motor progression of PD,^74^ and a genome-wide survival study this year identified a novel synaptic locus increasing the polygenic score of cognitive progression in PD.^75^ However, apart from large longitudinal prospective PD cohorts required, the input clinical quantitative measures and the algorithm to reflect the clinical progression of PD are also challenges in conducting such GWAS studies to truly reveal genetic factors associated with the progression of PD.

## Molecular pathways related to PD genetic factors

**Figure 1:**
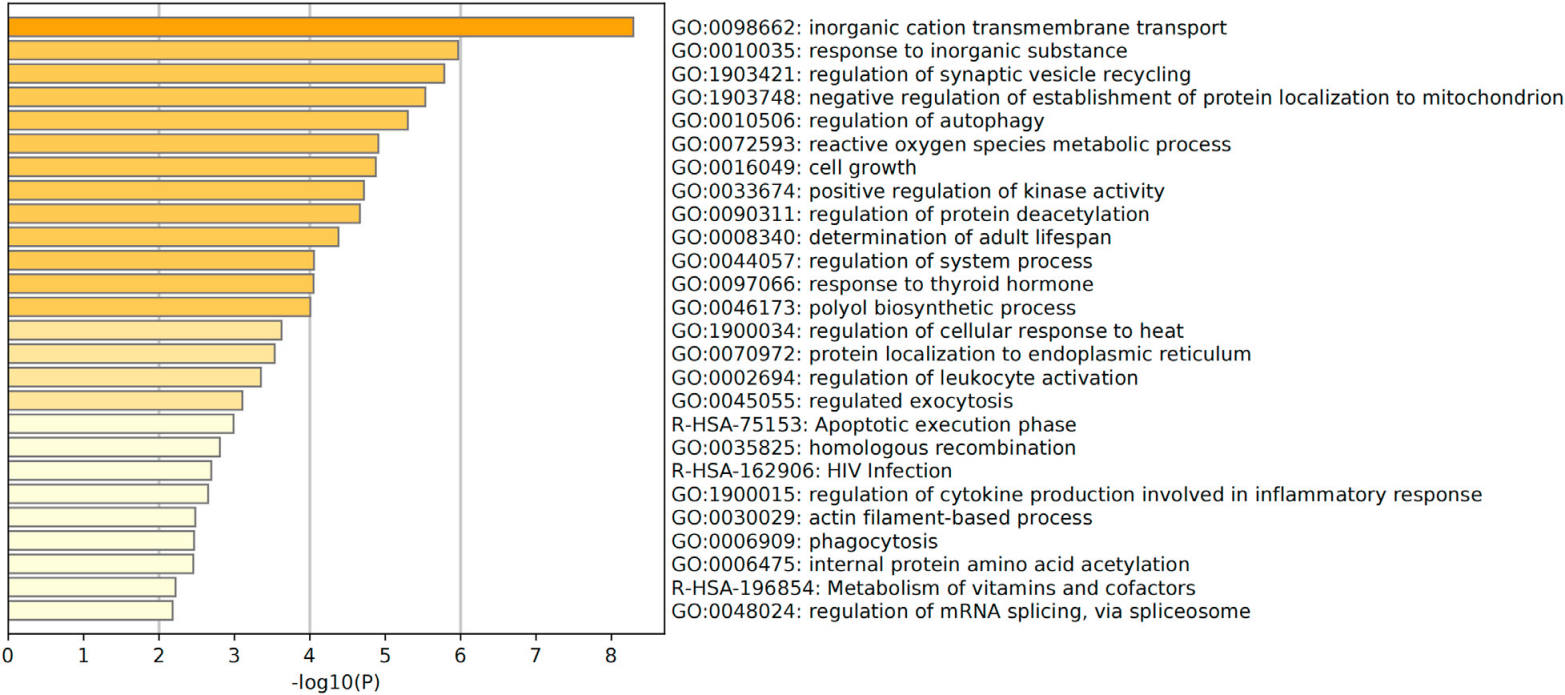
Bar chart of different categories of the protein functions. The proteins are encoded by the PD risk genes and their functions base on Gene Ontology (GO) database.^76^ The bar chart is stimulated using Metascape.^140^ The top 25 of the function categories were included in this chart.

The genes associated with PD risk are predominantly expressed in neurons,^65^ with some exceptions, e.g., Coetzee et al studied 4 risk loci containing risk SNPs which were shown in non-neuronal cells,^26^^,^^67^ encouraging future studies to investigate the function of SNPs associated with the etiology of PD. Majority of the associated genes of these SNPs are protein coding genes that shared the same biological pathways. There have been 10 biological pathways identified as enriched in these encoded proteins, 4 of which are associated with vacuolar functions, and three involving a known pharmaceutical target, e.g., kinase signaling and calcium transporters.^65^ According to the Gene Ontology (GO) database,^76^ the biological pathways of currently identified SNPs were listed and their associated genes belonging to different categories as demonstrated in Figure 1.

Figure 1 shows that the pathway with the highest *P*-value for enrichment of PD genes is inorganic cation transmembrane transport, a process whereby inorganic cations are transported across membrane by means of a transporter or pore. This is related to 712 genes, and 17 of them are related to PD, such as *LRRK2*, *RIMS1*, etc.^76^ In the PD risk gene products involved in inorganic cation transmembrane transport, 8 are associated with calcium ion transmembrane transport, which is important for regulation of mitochondrial function. Mitochondrial dysfunction and redox metals, i.e., inorganic substance with 12 genes involved, can cause oxidative stress, which has been shown to contribute to the etiology of PD.^77^ A recent study showed over-expression of α-synucleins increased Voltage Dependent Ion Channel 3 (VDIC3) permeability for calcium ions resulting in a net influx.^78^ Another cation, magnesium is also related to PD. Long-term magnesium deficiency leads to loss of dopaminergic neurons, and epidemiological studies show a higher incidence of PD in patients with low magnesium concentrations.^79^ These studies highlight the importance of inorganic cation imbalance to PD etiology, and as a potential target for therapeutic intervention.

**Figure 2:**
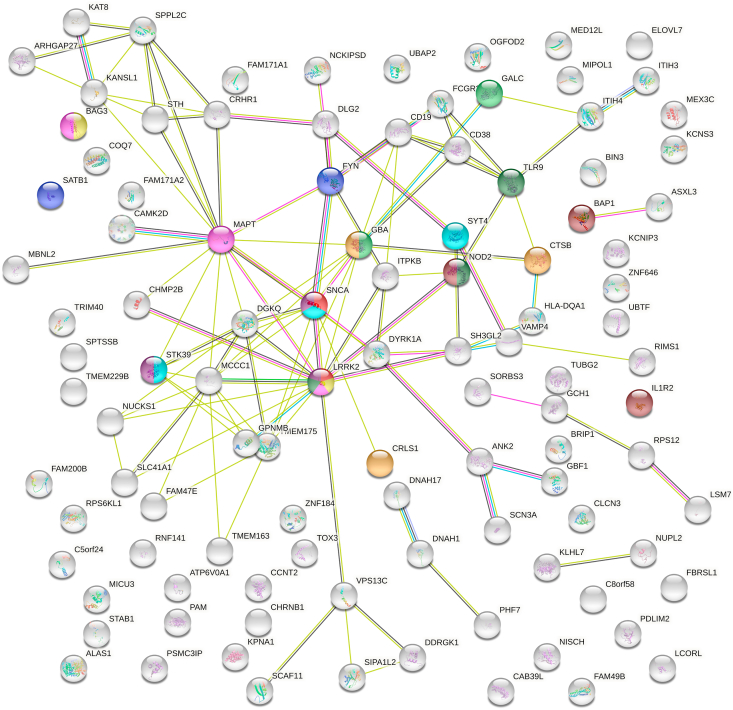
Network diagram demonstrates interactions between the risk gene products. The PD risk genetic products network shows 105 nodes and 111 edges. The different colours in the figure indicate different biological processes in which the highlighted genes are involved. Red represents “regulation of peroxidase activity”; Dark blue represents “activated T cell proliferation”; Light green represents “glycosylceramide catabolic process”; Light yellow represents “negative regulation of protein targeting to mitochondrion”; Pink represents “negative regulation of establishment of protein localization to mitochondrion”; Dark green represents “positive regulation of nitric-oxide synthase biosynthetic process”; Light blue represents “negative regulation of amine transport”; Dark yellow represents “response to thyroid hormone”; Purple represents “negative regulation of response to drug”; Brown represents “regulation of cytokine production involved in inflammatory response”.^76^ The graphic is made using STRING v11.0.

By STRING analysis (Fig. 2), PD risk genetic products network shows 105 nodes, of which 32 are hub nodes with connection degrees much greater than the average edge degree of the network which is 2.11.^80^ Some hub nodes demonstrate prominently abundant connections with other nodes, such as SNCA, LRRK2, MAPT, GBA, VPS13C, DGKQ and NOD2, which are all protein coding genes. Some of these are discussed above (Table 1). *DGKQ* encodes for diacylglycerol kinase θ protein, and is mainly expressed in the brain, mediating lipid and protein interaction in signal-transducing complexes,^81^ modulating calcium signalling and synaptic vesicles trafficking at nerve terminals.^82^ *NOD2* encodes nucleotide-binding oligomerization domain-containing protein 2, which are intracellular signalling proteins mediating NF-κB activation and apoptosis.^83^ Inflammation-derived oxidative stress accelerates the neurodegeneration in nigrostriatal pathway in PD.^83^

There are 111 edges in this network which is much greater than the expected number of 42, suggesting more interactions than random connections. Each edge represents a common pathway in which the genes products are involved. It demonstrates enrichment in certain biological process networks, such as regulation of peroxidase activity involving α-synuclein and LRRK2. These reflect the molecular pathways relevant to these genes involved in PD. The top 10 pathways with high strength of enrichment are colored in Figure 2, e.g., peroxidase regulation and activated T-cell proliferation. α-Synuclein and LRRK2 are involved in the peroxidase regulation pathway. Glutathione peroxidase has been shown to be protective against oxidative stress in the progression of PD.^84^ The activated T-cell proliferation pathway involves FYN and SATB1. The expanded terminal effector CD8^+^ and cytotoxic CD4^+^ peripheral T cells in PD patients^85^ suggest T-cells could be a therapeutic target to lessen neurodegeneration in PD.

Figure 2 reflects the molecular pathways relevant to these genetic products involved in PD, where reactive oxygen species induced inflammation/oxidative stress and mitochondrial dysfunction have also been shown in GO database analysis (Fig. 1). Interestingly, both analyses show that response to thyroid hormone is related to PD (Figure 1, Figure 2), which indicates that hypothalamic pituitary thyroid axis may play an important role in PD pathogenesis. Research has shown that regulation of thyroid-stimulating hormone and thyroid hormones correlates with the severity of PD.^86^

These identified pathways correlate with other databases including Reactome, KEGG, BIOCARTA, Pathway Interaction Database, Matrisome project, Signalling Gateway, Sigma Aldrich and SuperArray SABiosciences.^87^ These databases also suggest pathways such as lipid metabolism, immune response, synaptic transmission, endosomal–lysosomal dysfunction and apoptosis mediated by initiator and executioner caspases.^87^ Moreover, adaptive and innate immune response, vesicular-mediated transport, and lipid metabolism affected by signalling mechanisms were all associated with PD.^87^ Recent studies showed that LRRK2 phosphorylates SYNJ1 and DNAJC6 for vesicle endocytosis and recycling.^37^^,^^88^ Other PARK genes encoded proteins such as Parkin, involve AMPA-type glutamate receptor (AMPAR) trafficking.^89^ Mutations lead to AMPAR trafficking defects which affect synaptic plasticity, and this impacts on information processing leading to PD psychiatric symptoms.^8^^,^^90^ Further studies are required to identify other potential pathways and search for any link with the biological hallmarks of PD.

## Genetic implications for PD therapy

There have been several PD risk genes identified as therapeutic targets. The GBA target treatment focus on its encoded protein glucocerebrosidase, and glucosylceramide synthase inhibitor and ambroxol hydrochloride have already been used in clinical trials, with the latter therapy displaying promising indications.^91^ Early stage of clinical trials (BIIB094 and DNL201) targeting LRRK2 expression or its kinase activity are underway (NCT03976349 & NCT03710707). There is also evidence suggesting deep brain stimulation therapy is effective in certain monogenic PD patients such as those with *LRRK2* p.G2019S or *PRKN* mutations.^87^^,^^92^ This was not effective in patients with mutations such as *SNCA* and *GBA*, possibly due to their associated rapid disease progression.^92^

The identified genetic factors also contribute to the need to adjust the appropriate dosage of levodopa medication for PD patients. The mutations within genes involved in levodopa metabolism (*DDC* and *COMT*), dopamine transportation (*DAT*) and dopamine signaling (*DRD2* and *DRD3*) greatly affect the required dosage of these medications.^87^ Unfortunately, we still do not have sufficient insights as to what mechanisms to target in individual patients as we lack a full understanding of the pathways associated with PD. Therefore, identifying the genes with their biological pathway involved in current PD treatment regime is important in providing patients with personalized practice.

## Conclusion

Since the late 20th century, studies have been investigating genetic associations with PD. To date, there have been over 70 genes and their specific SNPs identified to increase PD risk. These genetic risk factors also associate with the type and severity of PD clinical manifestations, age of onset and PD progression. However, those identified so far only represent a small proportion of PD risk genetic factors. Future studies should continue exploring novel loci using advanced genotyping arrays in larger sample sizes. The heterogeneous genomic construct among different populations warrants validation and confirmation for PD susceptibility genes. In addition, characterizing the genomic contributions to the progression and subtypes of PD represents a medical advance poised to facilitate clinical practice in the real world of PD management. This would further increase the accuracy of disease treatment and provide a better management plan for PD patients, to achieve evidence-based, high-quality medicine.

## Author contributions

YH designed the project and critically revised the manuscript; JW drafted the manuscript; MM and AC co-supervised JW and actively participated in the manuscript revision.

## Conflict of interests

The authors declare no conflict of interests.

## Funding

This work is supported by 10.13039/501100001809National Natural Science Foundation of China (No. NSFC 82071417, YH). AC received grant funding from the Australian government.
